# The Reduction of Methane Production in the In Vitro Ruminal Fermentation of Different Substrates is Linked with the Chemical Composition of the Essential Oil

**DOI:** 10.3390/ani10050786

**Published:** 2020-05-01

**Authors:** Florencia Garcia, Darío Colombatto, M. Alejandra Brunetti, M. José Martínez, M. Valeria Moreno, M. Carolina Scorcione Turcato, Enrique Lucini, Georgina Frossasco, Jorge Martínez Ferrer

**Affiliations:** 1 Facultad de Ciencias Agropecuarias, Universidad Nacional de Córdoba, Córdoba X 5000, Argentina; eilucini@agro.unc.edu.ar; 2 Consejo Nacional de Investigaciones Científicas y Técnicas, Buenos Aires C1425FQB, Argentina; colombat@agro.uba.ar (D.C.); caroscorcione@gmail.com (M.C.S.T.); 3 Facultad de Agronomía, Universidad de Buenos Aires, Buenos Aires C1417DSQ, Argentina; 4 Estación Experimental Agropecuaria Manfredi, Instituto Nacional de Tecnología Agropecuaria, Córdoba 5988, Argentina; brunetti.alejandra@inta.gob.ar (M.A.B.); martinez.mariajose@inta.gob.ar (M.J.M.); moreno.maria@inta.gob.ar (M.V.M.); frossasco.georgina@inta.gob.ar (G.F.); martinez.ferrer@inta.gob.ar (J.M.F.)

**Keywords:** greenhouse gases, rumen fermentation, plant secondary metabolites, bioactive compounds

## Abstract

**Simple Summary:**

There is growing concern about how animal-derived foods are produced. Methane production in ruminants has received much attention in relation to its contribution to greenhouse gases and its effect on global warming. Another aspect of livestock production that is questioned by consumers is related to in-feed antibiotics added to improve feed efficiency, and due to health safety issues, their use has been banned or under revision in some parts of the world. Hence, there is the need to find new solutions to mitigate methane production in the rumen in a way that is considered safe and environmental-friendly by consumers and feasible, and without a negative impact on the farmers. Among the alternatives, the use of essential oils to modify rumen fermentation has attracted attention. This paper explores the effectiveness of essential oils obtained from two plants, *Lippia turbinata* and *Tagetes minuta*, to reduce methane production during the in vitro fermentation of substrates that are representative of different livestock production systems. The main conclusion to which we arrived is that the extent of the reduction in methane production depends on the interaction between the fermentation conditions that are generated by different substrates and the chemical profile of the essential oil, especially regarding its proportion of oxygenated compounds.

**Abstract:**

There is interest in identifying natural products capable of manipulating rumen microbial activity to develop new feed additives for ruminant nutrition as a strategy to reduce methane. Two trials were performed using the in vitro gas production technique to evaluate the interaction of substrate (n = 5) and additive (n = 6, increasing doses: 0, 0.3, 3, 30, and 300 µL/L of essential oils—EO—of *Lippia turbinata* or *Tagetes minuta*, and monensin at 1.87 mg/L). The two EO utilized were selected because they differ markedly in their chemical composition, especially in the proportion of oxygenated compounds. For both EO, the interaction between the substrate and additive was significant for all variables; however, the interaction behaved differently for the two EO. Within each substrate, the response was dose-dependent, without effects at a low level of EO and a negative outcome at the highest dose. The intermediate dose (30 µL/L) inhibited methane with a slight reduction on substrate digestibility, with *L. turbinata* being more effective than *T. minuta*. It is concluded that the effectiveness of the EO to reduce methane production depends on interactions between the substrate that is fermented and the additive dose that generates different characteristics within the incubation medium (e.g., pH); and thus, the chemical nature of the compounds of the EO modulates the magnitude of this response.

## 1. Introduction

There is strong pressure from a public opinion regarding the way in which the food we consume is produced. Although livestock represents an important function in several dimensions related to food security and territorial development [1], the perception that animal production has a negative effect on the environment is continuously growing. Among the aspects that generate pressure on the livestock sector is its contribution to the increase of greenhouse gases (GHGs). With this, livestock is pointed out as one of the factors responsible for climate change, which today is considered as one of the greatest challenges that human-kind must face [2]. Methane is the main GHG emitted by the livestock sector; it has a warming potential 28 times greater than carbon dioxide [3], and for the animal, represents also a loss of ingested energy [4]. 

The use of in-feed iohophore antibiotics (e.g., monensin) to improve feed efficiency is an extended practice in commercial farms. They selectively modulate the activity of some ruminal microorganisms [5], and results from a meta-analysis showed that monensin could reduce methane emissions and improve energy utilization in dairy and beef cattle [6]. However, due to the concern that pathogenic bacteria may develop resistance to antibiotics, using them for nutritional purposes is facing reduced acceptance by consumers [7]. Consequently, their use as a dietary additive has been limited or banned in some countries (e.g., in Argentina: Resolution SENASA 594/15 and 1119/18, in the European Union: Regulation 1831/2003/EC), and it is under revision in other nations. Thus, in the evaluation and selection of strategies to reduce methane production, there is the need to select those more acceptable to consumers and considered safer by international control agencies. In this regard, there is growing interest in the evaluation of natural additives with the potential to modify rumen fermentation.

Essential oils (EO) are complex mixtures of compounds from the secondary metabolism of plants, where they play an important protective role as antibacterial, antivirals, antifungals, and insecticides [8]. These compounds have diverse functional groups, such as aliphatic hydrocarbons, acids, alcohols, aldehydes, esters, ketones, and epoxides [8], which gives EO the ability to interact with diverse molecular and cellular objectives and trigger the response to the target [9,10]. Due to their antimicrobial activity, EO have been considered to provide a good opportunity for the development of natural additives capable of modulating rumen activity in a more “friendly” way with the environment [11], and thus, contribute to innovation in green technologies.

In previous work, two EO from plants native to Argentina, *Lippia turbinata* and *Tagetes minuta*, were identified to have great potential to favorably modulate ruminal microbial activity. In semi-continuous fermenters (Rusitec), the addition of 300 µL/L of either one of these EO decreased methane production by around 80% compared to the control, with no effect on volatile fatty acid concentration and composition, and a slight reduction on the substrate disappearance [12]. It has been previously reported that the bioactivity of EO depends, among other factors, on the substrate that is fermented [13,14]. The substrate used in that experiment was a feedlot-type diet, and then the question that arose was how these EO would perform in substrates that are representative of other diets. In general, in vitro studies are carried out based on one substrate, and there are few studies in which the effects of the addition of EO are evaluated in combination with different substrates in the same experiment [15,16,17].

The EO from *L. turbinata* exhibit bactericidal, fungicidal, and virucidal activity [18,19,20]. The component that was found to occur in the highest frequency in the *Lippia* oils is limonene [21]. The EO from *T. minuta* has a variety of uses, and it is known for its biocidic properties, acting against bacteria, fungi, and insects [22,23,24]. The composition of its oil is a distinctive characteristic of this group of plants, as it has components that are only present in this genus (e.g., dihydrotagetone, tagetone, and tagetenone) [25]. The antimicrobial potential of EO depends on the chemical composition of its active compounds, as well as their proportions and the interaction between their components, such as synergistic or antagonistic effects [26]. Thus, determining the chemical composition of EO in each experiment can provide a clear image of their overall potential as a feed additive [27]. 

This article proposes to deepen the characterization of the EO to find links between the composition of the additive used to manipulate rumen fermentation and the magnitude of the effect achieved in the fermentation of different substrates. Therefore, this study aimed to explore the effectiveness of EO of *L. turbinata* and *T. minuta* to reduce methane production using substrates that represent diets used in different livestock production systems. Substrates have different chemical composition and nutritional value; therefore, we hypothesized that the effects of EO on methane production would be of different magnitude depending on the fermentation characteristics that are created by incubating those substrates.

## 2. Materials and Methods 

The donor animals were managed following the guidelines and recommendations of the Ag Guide of the Federation of Animal Science Societies [28].

### 2.1. Experimental Design

The in vitro gas production technique described by Theodorou et al. [29] was used to evaluate the effect of increasing doses of EO of *L. turbinata* and *T. minuta* on methane production during the fermentation of different substrates. Two independent trials were carried out with the same methodology to evaluate each EO. Five substrates were evaluated, of which four typical simulated diets of livestock production systems—breeding, rearing, fattening, and dairy, while the remaining was a standard substrate that is used in our laboratory. The doses of EO were 0 (control), 0.3, 3, 30, and 300 µL/L. For each substrate, a treatment with monensin (Option 20%, Brascorp S.A., Buenos Aires, Argentina) at 1.87 mg of pure monensin/L was also included [30]. The EO doses and the monensin treatment were defined as additives. 

A factorial arrangement of treatments was carried out with the following two factors: substrate (n = 5) and additive (n = 6), summing up to a total of 30 treatments. A split-plot design was used, in which the main randomization factor (main plot) was the type of substrate, and the second factor (subplot) was the inclusion of the additive. Three incubations were performed in consecutive weeks, which were considered as blocks (repetitions). Within each block, three bottles were used for each treatment.

### 2.2. Essential Oils

The EO of *L. turbinata* and *T. minuta* were obtained by steam distillation of aerial parts of plants harvested at full flowering in Characato, Córdoba (31°28′77″ S, 64°12′32″ W). The chemical composition was analyzed, and it is described in Garcia et al. [12]. Briefly, the EO of *L. turbinata* had eleven compounds, with limonene as the main one (62%), followed by bornyl acetate (8%) and carvone (6%). Oxygenated terpenes accounted for less than 30% of the total composition, with hydrocarbons being the main functional group. The EO of *T. minuta* had nine compounds with four functional groups present; ketones were the most abundant (81%), followed by hydrocarbons (11%), alcohols (6%), and epoxides (2%). The main compounds present were verbenone (42%), *cis* tagetone (28%), limonene (6%), and *trans* tagetone (6%). Oxygenated terpenes accounted for almost 90% of the composition.

### 2.3. Substrate

The ingredients used to prepare the substrates were lyophilized and ground through a Wiley mill (2 mm screen). The proportions of the ingredients and the chemical composition of substrates are detailed in Table 1. The substrates were analyzed for the following parameters: organic matter (MO) by incineration at 550 °C for 4 h; crude protein (CP), performed by the Kjeldahl method (954.01, [31]); neutral detergent fiber (NDF) and acid detergent fiber (ADF), performed according to Van Soest et al. [32], using an Ankom 220 Fiber Analyzer unit (Ankom Technology Co., Fairport, New York, NY, United States), using heat-stable amylase and expressed inclusive of residual as, and in vitro dry matter digestibility (DMD*iv*), carried out according to Tilley and Terry [33], using a DaisyII incubator (Ankom Technology Co., Fairport, New York, NY, United States). 

### 2.4. In Vitro Incubation

On the day of the incubation and before the morning feeding, four liters of whole rumen content were collected in pre-warmed thermo-flasks from three rumen cannulated Hereford steers fed with alfalfa hay and corn grain (80:20 DM basis). Rumen content was pooled and homogenized in a blender for one minute, strained twice through two layers of cheesecloth, and the composited rumen fluid was collected in a flask. It was constantly homogenized using a magnetic stirrer, held at 39 °C in a water bath under CO_2_ stream until inoculation. The initial pH of the rumen fluid in the trial with *L. turbinata* was 6.54 ± 0.071 and in the trial with the EO of *T. minuta,* the initial pH was 6.50 ± 0.110. 

Incubations were carried out in 100 mL serum bottles, with 10 mL of rumen fluid, 40 mL of phosphate/carbonate buffer [33], and 0.5 g of the corresponding substrate mixture. Solutions of monensin and of EO were prepared in ethanol (70% v/v) at the necessary concentration to achieve the final dose in the bottle, and l mL of this solution was inoculated immediately before adding the rumen fluid. An equivalent amount of ethanol (70% v/v) was added to the control treatment bottles (dose 0). Blanks (bottles with rumen fluid and buffer, without substrate) were included so as to correct gas production and digestibility. Immediately after the rumen fluid was dispensed, the bottles were flushed with CO_2_ gas, sealed with butyl rubber stoppers and aluminum crimps, and placed in the water bath (39 °C).

Headspace pressure was measured at 1, 2, 3, 4, 6, 8, 12, 16, 20, 24, 30, 36, 42, and 48 h of incubation using a pressure transducer (Sper Scientific Ltd., Scottsdale, Arizona, United States) and monitored to prevent headspace pressure to reach 7.0 psi, as suggested by Theodorou et al. [29]. Gas was removed until the headspace pressure reached equilibrium (0.0 psi). The gas removed from one of the three replicates was collected using gas-tight syringes and accumulated into 250 mL vials for methane determination and stored at room temperature. Incubation bottles were manually shaken after each reading. At the end of the incubation (48 h), bottles were placed in ice to stop fermentation. 

### 2.5. Chemical Analyses, Determinations, and Calculations

#### 2.5.1. pH

This determination was performed only in the trial with the EO of *L. turbinata*. For this, one of the three replicates within each incubation (block) was open at the end of incubation, and pH was determined with a portable digital pH meter Sartorius PT-10 (Sartorius AG^®^, Göttingen, Germany). 

#### 2.5.2. Gas Production and Methane

A linear regression of pressure by volume (Volume (mL) = 4.905 × Pressure (psi) + 0.543), determined in previous studies, was used to obtain the volume of gas produced at each reading, which was corrected with the volume of gas produced by the blank. The accumulated net gas production was calculated as the sum of the corrected volumes at each measurement time. Gas production was determined from the three replicates, and data was averaged so as to have one value per block.

The methane concentration was determined by gas chromatography, using a Hewlett Packard 4890 (HP Inc., Palo Alto, CA, United States) equipped with a Porapak N 80/100 (2 m) analytical column with nitrogen as the carrier gas. The temperature of the injector was 110 °C, the column was held constantly at 90 °C during analysis, and the temperature of the flame ionization detector was 250 °C.

#### 2.5.3. Substrate Digestibility

The digestibility of the substrate was determined with the residues obtained at the end of incubation (48 h). One of the replicates was used to determine the organic matter digestibility (OMD), and the other replicate was used to determine the NDF digestibility (NDFD). The OMD was obtained by filtering the bottle content in pre-weighed crucibles (DURAN^®^ 25-851-32, DURAN Produktions GmbH & Co.; Hattenbergstr, Mainz, Germany). Dried residues were weighed after 24 h at 105 °C and ash determined by incineration at 550 °C for 4 h. The amount of substrate digested was calculated by the difference between the mass of incubated OM minus the mass of OM in the residue (after correction with the mass from the blank). The OMD was calculated as follows: OMD (g/g) = g OM digested/g OM incubated.

The NDFD was obtained according to Van Soest et al. [32]; briefly, the bottle content was transferred into 200 mL tubes, and 100 mL of neutral detergent solution plus heat-stable α-amylase were added. After boiling for 1 h, residues were filtered in pre-weighed glass crucibles (DURAN^®^ 25-851-32 filter crucibles) and oven-dried at 105 °C for 24 h. Dried residues were weighed and the ash determined by incineration at 550 °C for 4 h. The same procedure was carried out with the substrate to determine the mass of NDF incubated, and by the difference with the residues, the amount of NDF digested (after correction with the blank residue) was calculated. The NDFD was calculated as follows: NDFD (g/g) = g NDF digested/g NDF incubated.

#### 2.5.4. Values Relative to the Control

The relative gas and methane production, and the relative digestibility of the substrate (OMD and NDFD) due to increasing levels of EO compared to the control (dose 0) were calculated as follows: Relative production (%) = value with 0.3, 3, 30, or 300 µL/L of EO/value of 0 µL/L of EO) * 100.

### 2.6. Statistical Analysis

The data from both trials were analyzed independently with a mixed linear model to describe a split-plot design into a block arrangement using R language under the InfoStat software interface [34]. The mixed model accounted for the substrates (main plot; five levels), the additive (subplot; six levels), and their interaction as fixed effects. The blocks (n = 3) and main plots within the block were considered as random effects. The differences between treatments were evaluated with the multiple comparison test of means of Di Rienzo, Guzman, and Casanoves (DGC; α = 0.05) [35].

## 3. Results

### Effect of EO on Fermentation

The effect of increasing doses *L. turbinata* EO on total gas and methane production, and on the digestibility of the different substrates is presented in Table 2. The interaction of the substrate and additive was significant for all the variables under study, and within each substrate, the response was dose-dependent. At low doses (0.3 and 3 µL/L), no differences were detected on the variables under study, and the highest values of gas production and methane and digestibility were observed for the substrates representative of diets of fattening and dairy systems, and the lowest for the breeding substrate. With the intermediate dose (30 µL/L), gas and methane production was reduced, without affecting the digestibility of the substrates, except for a moderate decrease (−8.7%) of the OMD for the fattening substrate and a small reduction (−3.6%) of NDFD for the standard substrate. With the highest dose of EO (300 µL/L), the decrease in gas and methane production was notable but was associated with a marked decrease in substrates digestibility, with the exception of the OMD of the standard substrate. The effect of monensin was similar to that observed for the higher dose of EO (300 µL/L), which also reduced gas and methane production with a reduction in the digestibility of all substrates, except for OMD of the standard and rearing substrates. Regarding the effect of the different substrates at the same dose, at the low and intermediate levels of addition (0.3, 3, and 30 µL/L), all the variables evaluated were higher in the fattening substrate and lower in the breeding one. For the highest dose (300 µL/L), gas production and digestibility varied between the substrates, but no difference in methane production was observed.

**Figure 1:**
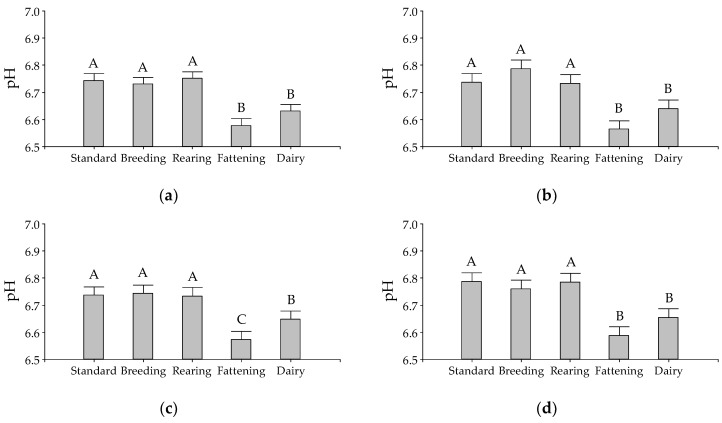

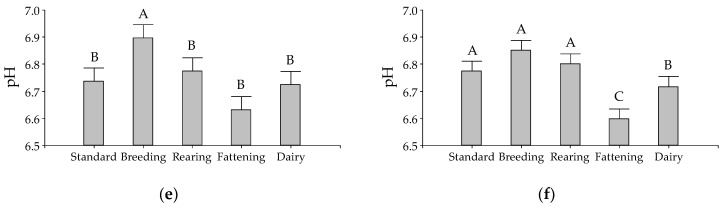
Effect of substrate on medium pH after 48 h in vitro incubation for different doses of essential oil of *Lippia turbinata*: (**a**) 0 µL/L (control); (**b**) 0.3 µL/L; (**c**) 3 µL/L; (**d**) 30 µL/L; (**e**) 300 µL/L; or (**f**) to the addition of 1.87 mg/L of monensin. The bars represent the standard error of the mean. ^A–C^ Different letters indicate statistically significant differences between substrates (Di Rienzo, Guzman and Casanoves (DGC), *p*-value < 0.05).

**Figure 2:**
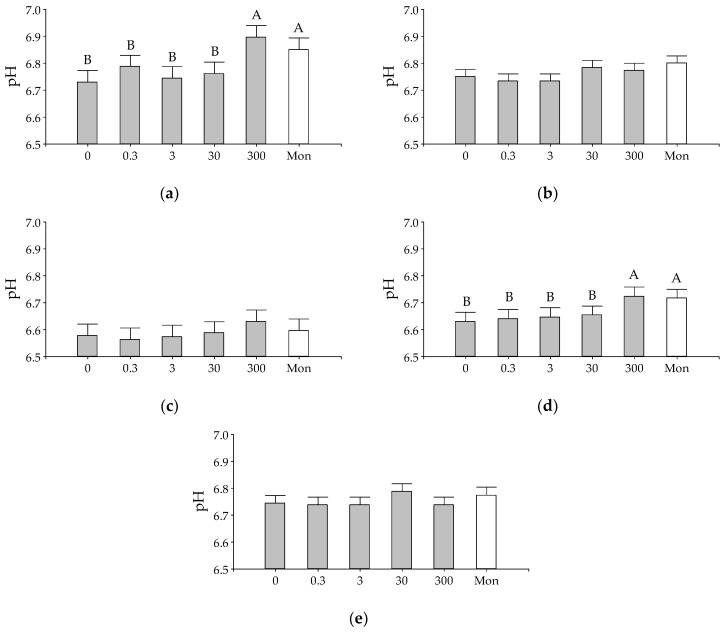
Effect of increasing doses (µL/L) of *Lippia turbinata* or the addition of 1.87 mg/L of monensin on medium pH after 48 h of in vitro incubation for different substrates: (**a**) breeding; (**b**) rearing; (**c**) fattening; (**d**) dairy, and (**e**) standard. The bars represent the standard error of the mean. In graphs (**a**,**d**), ^A,B^ different letters indicate statistically significant differences between substrates (DGC, *p*-value < 0.05).

The substrate and additive interaction were significant for pH after 48 h incubation (*p*-value = 0.0481). The effect of substrates within each additive on pH is shown in Figure 1. For all additives, except for the highest dose of EO (300 µL/L), the pH of the standard substrate, together with the substrates that simulated the breeding and rearing diets had higher pH values compared to the fattening and dairy substrates. The effect of additives within each incubated substrate on pH is shown in Figure 2. Only in the breeding and dairy substrates, the pH was increased by the addition of the higher dose of EO (300 µL/L) and monensin, with no other additive effect detected in pH for the rest of the substrates.

The effect of increasing doses of the EO of *T. minuta* on total gas and methane production, and on the digestibility of the different substrates is presented in Table 3. As with the EO of *L. turbinata*, the substrate and additive interaction was significant for all the variables under study; the highest values of gas and methane production and for digestibility were observed for the substrate representing a fattening system diet, and the lowest for the breeding substrate, and in all substrates the response was dose-dependent. At low doses (0.3 and 3 µL/L), no differences were detected in the variables studied compared to the control (dose 0). With the intermediate dose (30 µL/L), the production of gas and methane was reduced without affecting the digestibility in all substrates except the one that represents a fattening diet, in which no statistical difference was detected. With the highest dose of EO (300 µL/L), methane production was severely reduced, and no differences were observed between the different substrates, conversely, differences for the other response variables were still maintained at this higher dose. The effects due to monensin were similar to those observed for the trial with EO of *L. turbinata*.

**Figure 3:**
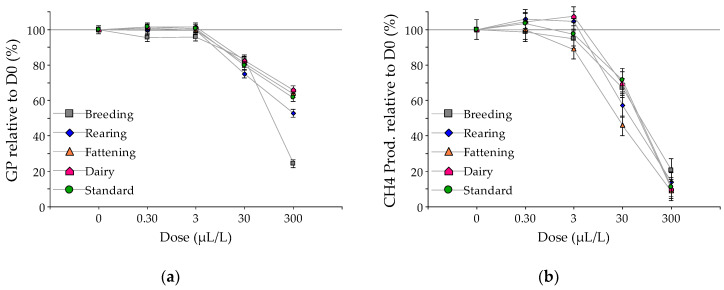

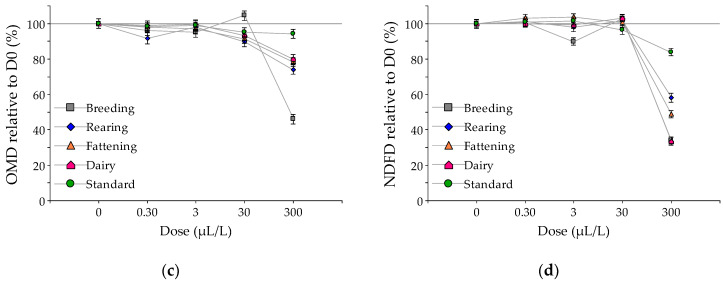
Relative responses compared to control (dose 0 = 100%) for (**a**) total gas production; (**b**) methane production; (**c**) organic matter digestibility (OMD) and (**d**) neutral detergent fiber digestibility (NDFD), to increasing doses of *Lippia turbinata* essential oil in the in vitro fermentation of substrates representing diets of different livestock production systems: breeding (gray), rearing (blue), fattening (orange), and dairy (pink), and a laboratory standard substrate (green).

**Figure 4:**
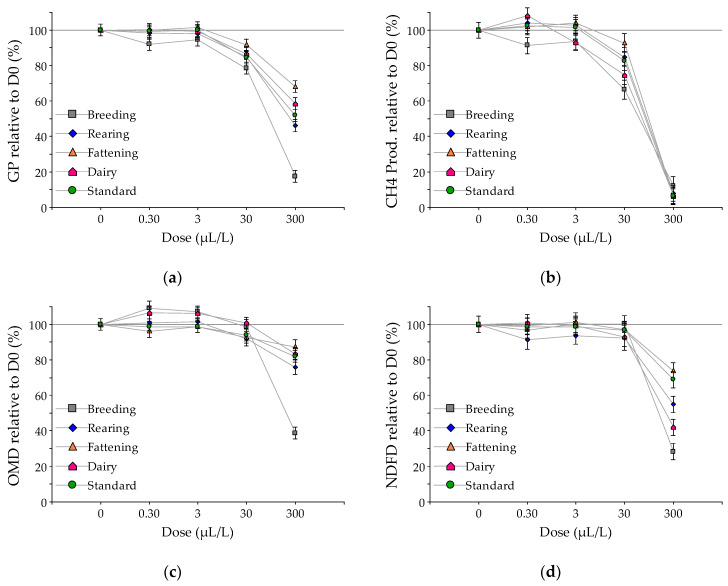
Relative responses compared to control (dose 0 = 100%) for (**a**) total gas production; (**b**) methane production; (**c**) organic matter digestibility (OMD) and (**d**) neutral detergent fiber digestibility (NDFD), to increasing doses of *Tagetes minuta* essential oil in the in vitro fermentation of substrates representing diets of different livestock production systems: breeding (gray), rearing (blue), fattening (orange), and dairy (pink), and a laboratory standard substrate (green).

Considering the control (dose 0) as reference (100%), Figure 3 and Figure 4 show the relative gas and methane production and the digestibility obtained by increasing the doses of EO of *L. turbinata* and *T. minuta*, respectively. With the intermediate dose (30 µL/L), the EO of *L. turbinata* reduced the total gas production by 20, 16, 25, 19, and 17% for the standard, breeding, rearing, fattening, and dairy substrates, respectively (*p*-value < 0.05). The reduction observed with the addition of *T. minuta* was 16, 22, 15, 8, and 13% for those substrates, respectively, (*p*-value < 0.05; except fattening that was not significant). At this level, the reduction of methane production ranged between 30 and 55% for the EO of *L. turbinata* (*p*-value < 0.01), while for *T. minuta* the maximum inhibition was 34% for the breeding substrate and the minimum was 8% for the fattening substrate (*p*-value < 0.05). At this intermediate dose, for both EO, neither OMD nor NDF digestibility were reduced, except a 7% reduction observed in OMD for the dairy substrate with *L. turbinata* EO (*p*-value < 0.05). With the highest dose (300 µL/L), the range of reduction of gas production was similar in both EO, being minimum in the fattening substrate (36 and 32% for *L. turbinata* and *T. minuta*, respectively) and maximum in the breeding substrate (76 and 83% for *L. turbinata* and *T. minuta*, respectively). At this high dose, the reduction in methane production was between 79 and 91% for *L. turbinata* and between 88 and 94% for *T. minuta*. The reduction in OMD for both EO was greater in the breeding substrate and lower in the fattening substrate, but to a different extent according to the EO. With *L. turbinate,* the reduction was 54 and 22%, respectively (*p*-value < 0.05), while for *T. minuta*, the reduction was 61 and 13%, respectively (*p*-value < 0.05). A similar effect was noted for fiber digestibility, where the reduction due to the addition of 300 µL/L of the EO of *L. turbinata* was 66, 51 and 66% for the breeding, fattening and dairy substrates, respectively (*p*-value < 0.05); while with *T. minuta*, the NDF digestibility was reduced by 72, 26, and 58%, respectively, for those substrates (*p*-value < 0.05).

## 4. Discussion

Essential oils are mixtures of numerous compounds of variable chemical identity, and their effectiveness as rumen fermentation modulators is strongly associated with their composition. In the present study, two EO with contrasting chemical composition were evaluated in vitro to assess the effect of increasing their doses on the fermentation of substrates of different nutritional values. Both EO have shown to be effective in modifying fermentation, and as expected, the effects depended both on the dose and on the incubated substrate; however, the interaction was different in the two EO. The variation in the behavior of the EO of *L. turbinata* and *T. minuta* for the substrates evaluated in this study is likely to be associated with the differences observed in their chemical composition, especially due to contrast in the proportion of oxygenated and non-oxygenated compounds.

The interaction of the substrate and additive was significant for all the variables studied in both EO, confirming that the effect of the additive is conditioned by the substrate on which the fermentation is performed [14]. It is expected that even from the same ruminal inoculum, in the in vitro incubation of substrates with different chemical composition, different microbial communities will develop and maintain active, and thus the fermentation products and the incubation conditions will also vary. Cardozo et al. [36] determined that one of the factors that may condition the effect of plant extracts on ruminal microbial fermentation is the pH of the medium, which may explain the differences observed with the addition of *L. turbinata* EO to the different substrates. We speculate that this also holds for the addition of *T. minuta* EO.

In all substrates, the effect of the addition of both EO depended on the dose, but the magnitude of the response varied among the two EO. At 30 µL/L, the EO of *L. turbinata* was more effective than *T. minuta* in reducing methane production in all substrates, with no negative effects on the digestibility of the substrates. With the highest dose (300 µL/L), the depressing effect of the EO of *L. turbinata* on the fiber digestibility in the fattening substrate was 1.51 times greater compared to that generated by the addition of the same dose of EO of *T. minuta*. A similar effect, but to a lesser extent (1.25 times), was observed with the digestibility of fiber in the dairy substrate. Conversely, in the breeding substrate, the EO of *T. minuta* reduced the digestibility of organic matter and fiber by 1.16 times more than when *L. turbinata* was added. In addition, only *T. minuta* reduced the OMD of the standard substrate. The fattening and dairy substrates were the ones with the greatest nutritional value, and as expected, they resulted in lower pH values of the fermentation medium at the end of the incubation at all levels of EO addition. Although this was only measured in the trial with *L. turbinata*, it could be expected that the same effect would have happened in the trial with *T. minuta*. If this speculation holds, the contrasting difference in the proportion of oxygenated compounds between both EO could be associated with the differences in response to the pH of the medium, generating the differences observed between the EO.

Although the mechanism of action of EO is not fully elucidated, one of the proposed sites of action is either the phospholipids’ bilayer [37] or within the cells [38]; therefore, the antimicrobial properties of EO are associated with its lipophilic character. Cardozo et al. [36] hypothesized that the difference in response of plant extracts according to pH might be related to the dissociated (hydrophilic) or non-dissociated (hydrophobic) state of the active molecules. If the mechanism of action is associated with the phospholipid membrane, only in the non-dissociated form of the molecules, the additives would be able to interact with the double layer of the cell membrane. When the pH drops, acids tend to be non-dissociated, and therefore, are more hydrophobic, so they interact more easily with cell membranes to generate their antimicrobial action. This could be true for *L. turbinata*, which showed greater activity on the fattening and dairy substrates, which were those that generated lower pH incubation medium values. On the other hand, the EO of *T. minuta* showed greater effect over the standard and the breeding substrates, in which medium pH is speculated to be higher, as those substrates presented a pH close to neutrality when assessed in the *L. turbinata* trial. 

The composition of *T. minuta* EO is a particular and distinctive feature of that genus [25]. The antimicrobial activity of EO of *T. minuta* has previously been attributed to the ketone functional group fractions [22,24], characterized by the presence of oxygen in the structure, which has been proposed as an element that increases the antimicrobial properties of terpenes [38]. The accounted proportions of verbenone, *cis* and *trans* tagetone and piperitenone are probably sufficient to be responsible for the observed antimicrobial activity. 

Among terpenoids, monoterpene hydrocarbons have shown the lowest antimicrobial activity, and even in some cases, they stimulate the rumen microbial activity [27]. On the contrary, the EO of *L. turbinata* used in the present study showed to be more effective than an EO rich in oxygenated compounds, even at a low dose (30 µL/L). Limonene was the main compound identified for *L. turbinata* (62%), and was also present in *T. minuta*, being the only component in common between both EO. In the present study, the doses of limonene added with the EO of *L. turbinata* were: 0.186, 1.86, 18.6, and 186 µL/L. Castillejos et al. [39] evaluated increasing doses of limonene (5, 50, 500, and 5000 mg/L) in ruminal in vitro fermentation (24 h) of a substrate similar in its chemical composition to the standard substrate used in the present study. In their study, limonene at 50 and 500 mg/L reduced the total concentration of volatile fatty acids (VFA), suggesting that at these doses, it is toxic to ruminal bacteria, while with the minimum dose (5 mg/L), the ruminal microbial fermentation was not affected. In a similar work, Castillejos [16] evaluated limonene in the same dose range, but on a substrate with a 10:90 forage:concentrate ratio, that had similar chemical composition to the dairy substrate used in the present study, and an increase in medium pH was reported for doses of 50 and 500 mg/L, without affecting other fermentation parameters. 

Similar to the present study, the addition of 300 mg/L of *Citrus aurantifolia* (51% limonene) reduced about 10% methane production during the in vitro fermentation of a tropical forage of low quality [40]. Burt [26] reported that there are interactions between the components of the EO, which may be of synergism or antagonism. Thus, their bioactivity may not be exclusively due to the presence of one or two components in greater proportion. While in the present study, the same dose of the EO of *L. turbinata* reduced methane production by 80% for the breeding substrate, indicating that other compounds in this EO may be bioactive or have a synergistic effect with limonene. Cattani et al. [41] evaluated the effects of limonene on methane production in an in vitro ruminal fermentation test using a substrate that simulated a dairy cow diet. Similar to the present study, the addition of 200 mg/L of limonene reduced the digestibility of dry matter and neutral detergent fiber, total gas and methane production, as well as ammonia concentration, and VFA production, and proportion. Joch et al. [42] evaluated limonene at 1000 µL/L in an in vitro experiment with a substrate composed of alfalfa silage, corn silage, and concentrate in a ratio of 35:35:30, and found a reduction in methane production without adverse effects on VFA production. In that study, the additives were inoculated directly into the incubation medium without prior dissolution. It is speculated that the effectiveness of EO may be increased when added with a solvent, such as ethanol, that can facilitate the dilution of the lipophilic fraction of the EO in the incubation medium, thus increasing its bioactivity [43]. The conditions of the fermentation medium, along with the interaction of limonene with other compounds present in the EO, could be the cause of the differences observed in the bioactivity of limonene.

Bornyl acetate was the second component in the highest proportion found in *L. turbinata* (8%). In the study by Joch et al. [42], bornyl acetate was defined as the most promising compound among the 11 EO compounds evaluated, which included different chemical groups, such as alcohols (linalool), aldehydes (citral), ethers (1,4 -cineol), hydrocarbons (p-cymene, limonene, α, and β pinene, γ terpinen), and phenolic compounds (carvacrol, eugenol). Bornyl acetate at 480 mg/L demonstrated the most beneficial effects on rumen fermentation when expressed as methane production per VFA production [44]. Although the dose was significantly higher than that of the present study, the lower relative activity of bornyl acetate at such high doses may be due to the fact that when added directly to the incubation medium and considering its lipophilic nature, the dispersion in the medium may have been smaller, thus reducing the toxicity of this compound to ruminal microorganisms. The antimicrobial activity of this compound may be related to the presence of the acetate portion in its structure [38], which increases the antimicrobial activity in comparison to the parent compound. The presence of bornyl acetate in the EO of *L. turbinata* may contribute to its bioactivity.

At the same dose and substrate, the reductions relative to the control (dose 0) were of greater magnitude for methane production than for total gas production, probably by selective suppression of methanogenic microorganisms [45,46,47]. In the same way, the addition of EO had a greater impact on the digestibility of the fiber, than on the digestibility of organic matter. Greater sensitivity to EO addition has been reported on populations of cellulolytic bacteria compared to other rumen microorganisms [45,46,47].

Compared to previous in vitro results in continuous cultures [12], the negative impact was greater in short fermentation systems than in Rusitec for the same dose (300 µL/L). In that study, although the digestibility decreased by 15%, no effects on the production and composition of VFA were detected, which are the main source of energy from ruminal fermentation for the host animal. The relationship between incubated DM and incubation volume differed in both in vitro systems; thus, the dose of 300 µL/L evaluated in the in vitro of short fermentation and in the Rusitec, was not the same when expressed on a substrate-incubated basis, being 30 and 12 µL/g DM, respectively. It has been pointed out that of the large number of studies that evaluated EO in in vitro fermentation experiments, only a few studies confirmed its effects in vivo [48]. The difficulty of translating the dose of one system to the following step might be a limitation scale-up in experimental complexity. For instance, Joch et al. [44] compared the effect of EO in vitro and in vivo, and observed that effects on fermentation in vitro were manifested at approximately 30 times higher concentrations than in in vivo conditions.

This study provided further evidence of the bioactivity of the EO of *L. turbinata* and *T. minuta*, demonstrating they are effective in modulating rumen fermentation in vitro of a variety of substrates. This study also confirms that the effect of EO depends on the substrate and the dose. In addition, it is proposed that the effectiveness of the EO to manipulate ruminal fermentation depends on interactions between the characteristics of the incubation medium (which depend on the substrate) and the chemical nature of the components of the EO, specifically in its proportion of oxygenated compounds. For a better understanding of how the fermentation environment modifies the physical and chemical properties of plant secondary metabolites, and by this their bioactivity, the evaluation of pure compounds would be of critical importance.

In vitro systems are a reduction of the rumen complexity that allow for a large number of treatments to be evaluated simultaneously [49]. This experimental approach is valuable for scanning purposes and to provide useful information to explain what might be happening in the rumen; however, the ultimate goal of this study is the development of an additive to modify rumen microbial activity. From the results of this study, we can conclude that the EO of *L. turbinata* and *T. minuta* can be considered as candidates for the development of new additives in ruminant nutrition as a strategy to mitigate the production of enteric methane. For its potential applicability, in vivo evaluations are critically necessary to confirm the effects on the animals and to optimize the dose according to the production system in which they are to be applied. Likewise, this approach will allow evaluating the persistence of the effects and the possible transfer of components and/or derived metabolites that may affect the quality of meat or milk produced.

## 5. Conclusions

The addition of essential oils of *Lippia turbinata* and *Tagetes minuta* to the incubation medium was effective in reducing methane production during the in vitro fermentation of a range of substrates that represent different livestock production systems. The magnitude of the antimethanogenic effect is affected by the type of EO, and it is substrate- and dose-dependent.

It is of interest to continue with the study of these EO as natural additives for methane reduction in ruminants, for which it would be necessary to confirm their effects in in vivo experiments.

## Figures and Tables

**Table 1:** Ingredients and chemical composition of the experimental substrates.

Item	Standard	Breeding	Rearing	Fattening	Dairy
Ingredients (%, DM basis)					
Alfalfa hay	80	-	15	15	15
Corn grain	20	-	-	80	-
Wet corn grain	-	-	-	-	28
Corn silage	-	-	-	-	46
Sorghum silage	-	-	70	-	-
Soybean expeller	-	-	15	5	10
*Panicum maximum*	-	100	-	-	-
Urea	-	-	-	-	1
Chemical composition (g/kg DM)					
Organic matter	913	897	910	968	946
Crude protein	178	40	167	124	164
Neutral detergent fiber	373	770	472	175	265
Acid detergent fiber	239	462	281	71	142
In vitro dry matter digestibility	739	554	681	877	821

**Table 2:** Effects of increasing doses of essential oil of *Lippia turbinata* or monensin addition on total gas and methane production and organic matter and fiber digestibility during the in vitro fermentation of substrates, representing different livestock production system diets and a laboratory standard substrate.

Substrate	Additive ^1^	Gas Production mL/g OM	Methane Production mL/g OM	OMD ^2^ g/g	NDFD ^3^ g/g
Standard	0	289 ^aB^	34.3 ^aA^	0.662 ^aC^	0.485 ^aC^
	0.3	293 ^aB^	35.5 ^aA^	0.648 ^aB^	0.489 ^aC^
	3	290 ^aB^	33.1 ^aA^	0.655 ^aB^	0.492 ^aB^
	30	230 ^bB^	24.3 ^bA^	0.631 ^aB^	0.470 ^bC^
	300	178 ^cB^	3.7 ^cA^	0.624 ^aA^	0.407 ^cA^
	Monensin	223 ^bB^	20.2 ^bA^	0.636 ^aB^	0.456 ^bA^
Breeding	0	199 ^aD^	25.2 ^aB^	0.420 ^aE^	0.441 ^aD^
	0.3	190 ^aD^	24.8 ^aC^	0.404 ^aD^	0.443 ^aD^
	3	191 ^aD^	23.4 ^aB^	0.398 ^aD^	0.396 ^aD^
	30	167 ^bC^	16.8 ^bA^	0.439 ^aD^	0.454 ^aC^
	300	49 ^dD^	5.2 ^cA^	0.193 ^bC^	0.149 ^bB^
	Monensin	74 ^cD^	7.3 ^cC^	0.242 ^bD^	0.180 ^bC^
Rearing	0	243 ^aC^	29.1 ^aB^	0.581 ^aD^	0.421 ^aD^
	0.3	242 ^aC^	30.7 ^aB^	0.532 ^aC^	0.426 ^aD^
	3	243 ^aC^	30.5 ^aA^	0.569 ^aC^	0.412 ^aD^
	30	182 ^bC^	16.3 ^bA^	0.521 ^aC^	0.427 ^aD^
	300	128 ^dC^	3.9 ^cA^	0.430 ^bB^	0.245 ^cB^
	Monensin	169 ^cC^	15.3 ^bB^	0.519 ^aC^	0.347 ^bB^
Fattening	0	332 ^aA^	37.8 ^aA^	0.891 ^aA^	0.709 ^aA^
	0.3	334 ^aA^	37.6 ^aA^	0.873 ^aA^	0.731 ^aA^
	3	330 ^aA^	33.7 ^aA^	0.864 ^aA^	0.735 ^aA^
	30	269 ^bA^	17.2 ^bA^	0.814 ^bA^	0.713 ^aA^
	300	211 ^cA^	3.3 ^cA^	0.698 ^cA^	0.346 ^cA^
	Monensin	259 ^bA^	17.0 ^bB^	0.813 ^bA^	0.463 ^bA^
Dairy	0	303 ^aB^	34.4 ^aA^	0.811 ^aB^	0.592 ^aB^
	0.3	307 ^aB^	35.5 ^aA^	0.801 ^aA^	0.591 ^aB^
	3	308 ^aB^	36.7 ^aA^	0.806 ^aA^	0.588 ^aB^
	30	250 ^bA^	22.9 ^bA^	0.756 ^bA^	0.610 ^aB^
	300	200 ^cA^	3.4 ^dA^	0.648 ^cA^	0.197 ^cB^
	Monensin	209 ^cB^	14.5 ^cB^	0.709 ^bB^	0.278 ^bB^
Standard error		6.9	1.77	0.0281	0.0186
*p*-value	Substrate (S)	<0.0001	0.0001	<0.0001	<0.0001
	Additive (A)	<0.0001	<0.0001	<0.0001	<0.0001
	S × A	<0.0001	0.0008	0.0002	<0.0001

^a–d^ Different lowercase letters in the same column indicate differences between essential oil (EO) doses or monensin within the same substrate (Di Rienzo, Guzman and Casanoves (DGC), *p* < 0.05). ^A–E^ Different capital letters in the same column indicate differences between substrates for the same EO dose or monensin (DGC, *p* < 0.05). ^1^ Essential oil dose expressed in µL/L. ^2^ OMD: Organic matter digestibility; ^3^ NDFD: Neutral detergent fiber digestibility.

**Table 3:** Effects of increasing doses of essential oil of *Tagetes minuta* or monensin addition on total gas and methane production and organic matter and fiber digestibility during the in vitro fermentation of substrates, representing different livestock production system diets and a laboratory standard substrate.

Substrate	Additive ^1^	Gas Production mL/g OM	Methane Production mL/g OM	OMD ^2^ g/g	NDFD ^3^ g/g
Standard	0	287 ^aB^	33.5 ^aA^	0.680 ^aB^	0.430 ^aB^
	0.3	284 ^aB^	34.4 ^aB^	0.670 ^aB^	0.422 ^aC^
	3	286 ^aB^	34.0 ^aA^	0.670 ^aC^	0.420 ^aC^
	30	244 ^bB^	27.9 ^bA^	0.640 ^aB^	0.414 ^aB^
	300	149 ^cB^	2.2 ^dA^	0.557 ^bB^	0.297 ^cB^
	Monensin	211 ^bB^	18.4 ^cA^	0.633 ^aB^	0.351 ^bB^
Breeding	0	193 ^aC^	25.9 ^aB^	0.344 ^aC^	0.365 ^aB^
	0.3	189 ^aC^	23.5 ^aD^	0.372 ^aC^	0.354 ^aC^
	3	195 ^aC^	24.1 ^aB^	0.353 ^aD^	0.366 ^aC^
	30	162 ^bC^	17.1 ^bB^	0.337 ^aD^	0.364 ^aB^
	300	36 ^dC^	02.3 ^dA^	0.128 ^bC^	0.104 ^bB^
	Monensin	59 ^cD^	7.1 ^cB^	0.138 ^bD^	0.103 ^bC^
Rearing	0	251 ^aB^	28.2 ^aB^	0.617 ^aB^	0.497 ^aB^
	0.3	247 ^aB^	29.1 ^aC^	0.615 ^aB^	0.465 ^aC^
	3	246 ^aB^	28.8 ^aB^	0.606 ^aC^	0.456 ^aC^
	30	215 ^bB^	23.8 ^bB^	0.562 ^aC^	0.452 ^aB^
	300	116 ^dB^	2.2 ^dA^	0.462 ^bB^	0.264 ^bB^
	Monensin	167 ^cC^	14.4 ^cA^	0.545 ^aC^	0.292 ^bB^
Fattening	0	347 ^aA^	35.8 ^aA^	0.873 ^aA^	0.818 ^aA^
	0.3	347 ^aA^	36.5 ^aB^	0.839 ^aA^	0.811 ^aA^
	3	352 ^aA^	37.2 ^aA^	0.862 ^aA^	0.825 ^aA^
	30	319 ^aA^	33.3 ^aA^	0.808 ^aA^	0.794 ^aA^
	300	237 ^cA^	2.2 ^cA^	0.757 ^bA^	0.608 ^bA^
	Monensin	285 ^bA^	18.7 ^bA^	0.828 ^aA^	0.676 ^bA^
Dairy	0	385 ^aA^	39.3 ^aA^	0.710 ^aB^	0.542 ^aB^
	0.3	386 ^aA^	41.8 ^aA^	0.753 ^aA^	0.545 ^aB^
	3	381 ^aA^	36.8 ^aA^	0.750 ^aB^	0.537 ^aB^
	30	331 ^bA^	29.4 ^bA^	0.715 ^aB^	0.486 ^aB^
	300	225 ^dA^	2.7 ^dA^	0.593 ^bB^	0.234 ^bB^
	Monensin	276 ^cA^	16.4 ^cA^	0.650 ^bB^	0.267 ^bB^
Standard error		15.4	2.65	0.0279	0.0420
*p*-value	Substrate (S)	<0.0001	0.0001	<0.0001	0.0001
	Additive (A)	<0.0001	<0.0001	<0.0001	<0.0001
	S × A	0.0338	0.0012	0.0002	0.0114

^a–d^ Different lowercase letters in the same column indicate differences between essential oil (EO) doses or monensin within the same substrate (DGC, *p* < 0.05). ^A–D^ Different capital letters in the same column indicate differences between substrates for the same EO dose or monensin (DGC, *p* < 0.05). ^1^ Essential oil dose expressed in µL/L. ^2^ OMD: Organic matter digestibility; ^3^ NDFD: Neutral detergent fiber digestibility.
